# Bacterial flora of combat wounds from eastern Ukraine and time-specified changes of bacterial recovery during treatment in Ukrainian military hospital

**DOI:** 10.1186/s13104-017-2481-4

**Published:** 2017-04-07

**Authors:** Kovalchuk P. Valentine, Kondratiuk M. Viacheslav

**Affiliations:** 1 Department of Microbiology, Vinnitsa National Medical University, Pirogova str 56, Vinnitsa, 21018 Ukraine; 2 Military Medical Clinical Center of Central Region, Sverdlova str 185, Vinnitsa, 21018 Ukraine

**Keywords:** War wounds, Microbial flora, Hybrid war in eastern Ukraine

## Abstract

**Background:**

Microbiology of modern war wounds is unique for each military conflict. Climatic and geographical features of the theater of war, contemporary warfare as well as wound management affect the microbial flora of wounds. This study was designed to determine time-specific microbial flora of combat wounds of upper and lower extremities obtained during the war in eastern Ukraine.

**Methods:**

The patients enrolled in study had combat wounds of upper or lower extremities which were treated in the Military Medical Clinical Center of Central Region. The wounds were swab-cultured and measured at each surgical debridement. The recovered microorganisms were identified and their antimicrobial resistance profiles were evaluated by disc diffusion method.

**Results:**

Forty-nine patients with battle-field wounds were enrolled in the study from July to November 2014; all patients were male with a mean Injury Severity Score and arrival APACHE II scores of 16.2 ± 10.7 and 7.4 ± 4.2 respectively. Among 128 swab cultures, 100 swab cultures were positive. Swab cultures were obtained from 57 wounds of 49 patients. The results of the test showed that 87.7% of all positive swab cultures contained a single-organism while the rest of the swab-culture results showed polymicrobial growth. Among the isolated microorganisms 65% (76 strains) were Gram-negative rods, 22.2% (26 strains) of Gram-positive cocci, followed by Gram-positive rods (12.8%, 15 strains). We found that epidemiology of wound infection changes with the time after injury. The most common bacterial isolates cultured during the first week were Gram-positive microbes with low pathogenicity. The number of Gram-negative rods increased during the wound healing process. The incidence of Gram-positive microorganisms’ growth fell after the first week and increased after third week. During wound healing, bacterial microflora of wounds changes with increasing number of Gram-negative rods with predominance of *Acinetobacter* species. Predominant microorganisms in positive swab-cultures after first week were nonfermentative Gram-negative bacilli (68% of swab-cultures), which in 53% of the swab-cultures belonged to the genus *Acinetobacter*, and in 15% to the genus *Pseudomonas*. The incidence of polymicrobial wound cultures increased from first week to second post-injury week. The most frequent microbial mixture were *Acinetobacter baumannii* with *Enterobacteriaceae* or other nonfermentative Gram negative rods with *Enterococcus* spp. We observed bacteria recovery from wounds during proliferation phase. These wounds had no pure inflammation signs and were free of devitalized tissues.

**Conclusions:**

Any wound is at some risk of becoming infected. In the event of infection, a wound fails to heal, treatment costs rise, and general wound management practices become more resource demanding. Determining the microorganisms which colonize battle wounds and cause wound infection is paramount. This information can help to treat battle wound infections or even changes infection control strategies. The fact of shifting in wound microbiology in the favor of bacteria responsible for healthcare-associated infections support to the proposition that these changes are nosocomially related [4, 14]. For Ukrainian military medicine this study is the first time-specified assessment of battle wound colonization from the World War II.

**Electronic supplementary material:**

The online version of this article (doi:10.1186/s13104-017-2481-4) contains supplementary material, which is available to authorized users.

## Background

The microbiology of battle wound has changed as medicine and warfare have evolved. There are significant differences in the microflora of wounds from different war conflicts. Relative abundance of major bacterial groups differ during wound healing [1]. Initial wound-colonizing bacteria are low-virulence environmental contaminants that do not result in sustained infections under the treatment conditions. The microbiology of US casualty soft tissues vary as casualties pass through the current military medical system from combat theater and interim echelon facilities to final echelon care [2]. Colonization with resistant microorganisms increased during evacuation through the various levels of the evacuation chain. These infections are thus likely the result of hospital-acquired infection in facilities along the evacuation route [3]. A Gram-negative bacteria like *Enterobacter aerogenes*, *Klebsiella pneumoniae*, *Pseudomonas* species, *Proteus* species and *Escherichia coli* predominated throughout the studies, with *Acinetobacter baumannii* being the most common pathogen [1]. *Acinetobacter* appears to be primarily associated with nosocomial transmission in and out of the combat zone [4]. However, the structure of the etiologic agents of wound infection in each hospital varies considerably and cannot be predicted exactly. One of the most disconcerting facts about the bacteria complicating combat casualties is their increasing antimicrobial resistance [5].

For these reasons microbiology of the wounds acquired in modern wars is unique for each military conflict. Climatic and geographical features of the theater of war as well as accessible wound management affects on the microbiology of war wounds obtained in eastern Ukraine. The specific of the hybrid warfare in Ukraine create new unique environmental conditions for military medical system. The present study was designed to determine time-specific microbial flora of battle-field wounds of upper and lower extremities caused during the war in eastern Ukraine.

The study was conducted in Military Medical Clinical Center of Central Region (MMCC CR) located in Vinnitsa. This is one of the five Ukrainian Military Medical Clinical Centers equal to NATO level IV hospital. Here definitive surgical treatment for wounded patients was provided. Wounded passed through several medical facilities before arriving here. The Ukrainian military medical system was distributed into four tiers. NATO ROLE I to III hospitals were deployed in the theater of operations. A lot of civilian hospitals with the help of military surgeons work as NATO ROLE II hospital, providing care limited to damage-control surgery. In ROLE III military combat support hospitals wounded patients were treated up to 15 days and then were evacuated to any Military Medical Clinical Centers for definitive treatment. The medical evacuation process was complicated, and more than a week may be spent evacuating patients through two to four facilities where they receive care. Each hospital has varying management and infection control strategies.

## Methods

### Ethical considerations

Hospital permission to conduct the surveillance study was obtained from Hospital Bioethics Committee of MMCC CR, Ministry of Defense, Vinnitsa, Ukraine, protocol reference number 18/2 from 05 May 2014. The patients were included after understanding the study and had signed an informed consent.

### Patient population

During July–November 2014, Ukrainian military casualties arrived at MMCC CR located in Vinnitsa (NATO ROLE IV), with an acute traumatic injury resulting in an open wound were eligible for enrollment into study of battle wound microbial flora. Eligible patients were adult male military with extremity wounds sustained in the east of Ukraine (without communication to the thoracic or abdominal cavities). All wounds were treated with repeated surgical debridement, vacuum assistance closure (VAC) and wound bed preparation. Up to two wounds maximum per patient were evaluated. In patients with more than two eligible wounds, the largest were evaluated. Wounds of 44 patients were ultimately closed by either delayed primary closure or a split-thickness skin graft at the discretion of the patient’s attending surgeon, five patients were transferred to another hospital before final healing. Data collected for each casualty at the time of arrival included demographic variables, injury specifics with wound characteristics, previous field medical care (including wound management), antimicrobial use and clinical parameters with Injury Severity Score and APACHE II scoring. During the course of care wounds were photographed and dimensions were measured before each surgical debridement. Wound margins were traced onto a sterile transparent film, and the area was calculated by computerized planimetrics [6]. Wound surface area (cm^2^) and volume (cm^3^) were calculated. Surgical debridement, pulse lavage, and VAC reapplication were performed every 48–96 h. For 48 wounded (98%) VAC was started only at MMCC CR. Only black VAC sponges were used during the study.

### Culture technique

Two culture swabs (Culture Swab collection and transport system for aerobes and anaerobes; SARSTEDT AG & Co Germany) were inserted into the wounds during surgical wound bed preparation and were then directly transported to the laboratory. Swabs were plated on sheep’s blood agar and MacConkey plates in triplicate and then were left in thioglycolate broth. Blood agar plates and MacConkey agar plates were incubated at 35 °C in ambient air. Cultures were held for up to 5 days before results were classified as negative. Phenotypic identification of colonies was accomplished using automated bacterial identification system (VITEK^®^ 2 Compact 30 BioMérieux’s). Microbial sensitivity testing was done using disk diffusion test as per EUCAST (European Committee on Antimicrobial Susceptibility Testing) guidelines [7]. Antibacterial agents to be tested for each organism were selected based on hospital antibiotic policies.

## Results

Forty-nine patients were enrolled in the study from July to November 2014; all patients were male. Thirty-nine patients had 1 wound, 8 patients had 2 wounds for a total of 57 wounds. All patients were male with a median age of 30.4 ± 8.9 years with a mean Injury Severity Score and arrival APACHE II scores of 16.2 ± 10.7 and 7.4 ± 4.2 respectively. No patients had evidence of traumatic brain injury or other diseases caused immunosuppression. Twenty-two patients (44.8%) had blast injury (39 wounds), 18 wounds were caused by gunshots (16 casualties) (Table 1). During pre-ROLE IV care surgical debridement were performed and antibiotics were administered for all wounded. The data about numbers of debridement and the number of antibiotics administered were inconsistently available/verifiable.

**Table 1:** Patient demographics

Characteristics		Percentage (%)
Age	30.4 ± 8.9	
Injury Severity Score	Mean 16.2 ± 10.7	
APACHE II score (at arrival)	Mean 7.4 ± 4.2	
Wounds per patient		
1 wound	41	83.7
2 wound	8	16.3
Time from injury to arrival at VMMC CR, days	Mean 5.2 ± 4.6	
The mean number of echelons passed	2.9 ± 0.56	
The mean number of surgical debridement	1.9 ± 0.56	
Mechanism of injury (wounds)		
Blast	39	68.4
Gunshot	18	31.6
Acute traumatic injury resulting in		
Open fractures		
Type II	2	3.5
Type III A	5	8.8
Type III B	24	42.1
Type III C	11	19.3
Isolated soft tissue injury	15	26.3
Wound location		
Upper limb	16	28.1
Lower limb	41	71.9

**Fig. 1:**
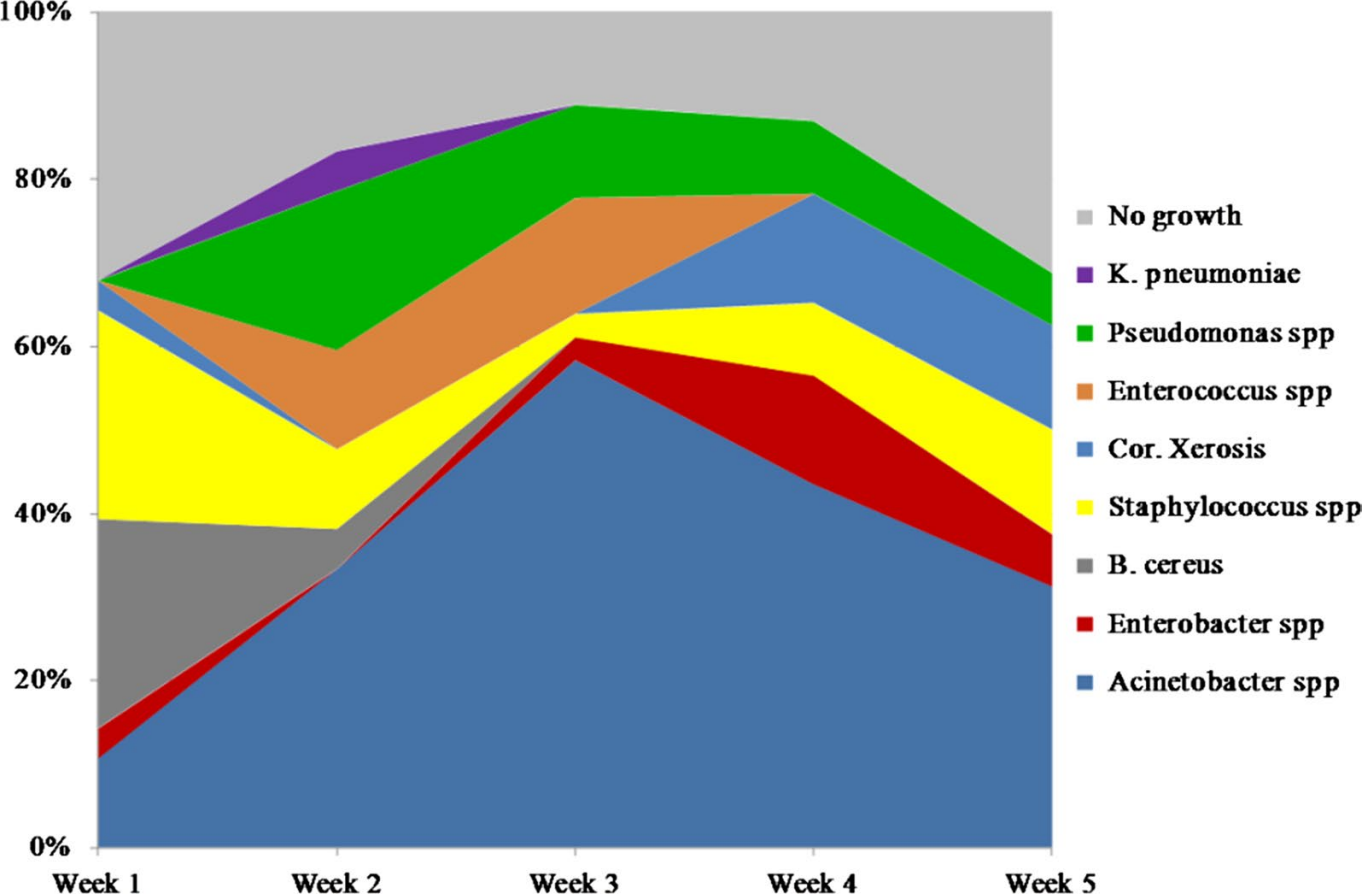
A proportion of microorganisms along wound healing on a week basis. A graph demonstrates the difference in wound microbial community composition from week to week over a 5-week period

Fifty-seven wounds were studied and included in the analysis. Forty-one wounds (71.9%) were in the lower extremities and 16 in the upper extremities (28.1%). The mean surface area of the wounds was 95.6 cm^2^ [standard deviation (SD) ±123.7 cm^2^, range 5.1–452 cm^2^]. The mean wound volume was 456.2 cm^3^ (SD ±853 cm^3^, range 11.5–3740.8 cm^3^). One hundred twenty-eight swab cultures were performed on the 57 wounds. The mean number of swab culture per wound was 2.5 (SD ±0.8). Of the 128 swap cultures, 28 cultures (21.8%) showed no growth and 100 (78.1%) were positive for bacterial growth (Fig. 1).

One hundred positive swab cultures were obtained from 57 wounds of 49 patients. The results of the test showed that 87.7% of all positive swab cultures contained a single-organism while the rest of the swab-culture results showed polymicrobial growth. Among the isolated microorganisms 65% (76 strains) were Gram-negative rods, 22.2% (26 strains) of Gram-positive cocci, followed by Gram-positive rods (12.8%, 15 strains).

Predominate microorganisms in positive swab-cultures were nonfermentative Gram-negative bacilli (68% of swab-cultures), which in 53% of the swab-cultures belonged to the genus *Acinetobacter*, and in 15% to the genus *Pseudomonas*. Microorganisms family *Enterobacteriaceae* isolated from 8% of positive swab-cultures. Six swab-cultures revealed six strains belonged to the genus *Enterobacter*, two swab-cultures revealed strains belonged to the genus *Klebsiella*. Gram-positive cocci were isolated in 24% of positive swab-cultures. The incidence of genus *Staphylococcus* isolation were 13% (13 strains) followed by *Enterococcus* genus (10% of positive swab-cultures revealed 10 strains). However, only small amount of *Staphylococcus* spp. (2 strains, 2% of positive swab-cultures) were coagulase-positive. Coagulase-negative staphylococci were identified as *S. epidermidis* (10 strains) and as *S. haemoliticus* (3 strains). The incidence of any bacterial isolation from biopsies weekly was 67.9, 80, 87.5, 84.2 and 64.3%, respectively. Polymicrobial tissue cultures occurred at following incidences 7, 17.1, 9.3, 21 and 14.3%.

The predominant flora in wounds changes with time after injury. Within first week of an injury 28 swab cultures were taken from 15 wounded, but only 9 swabs showed no growth (32.1% of swab-cultures*).* The main microorganisms were Gram-positive bacteria with predominance of *S. epidermidis* (7 strains, 36.8% first week positive swab-cultures) and *Bacillus cereus* (7 strains, 36.8% first week positive swab-cultures). Gram-negative rods included the following: *A. baumannii* (3 strains, 15.8% first week swab-cultures) and one case *Enterobacter cloacae* ssp. cloacae.

During second week 35 swab culture were taken from 24 wounded patients. Only seven (20%) swab culture in this term had no growth. Eleven swab-cultures (31.4%) revealed 11 Gram-positive microorganisms. With this, the most common isolates were *Enterococcus* spp. (5 strains, 17.9% second week positive swab-cultures), followed by coagulase-negative staphylococci (4 strains, 14.3% second week positive swab-cultures). The incidence of nonfermentative Gram-negative bacilli and rods family *Enterobacteriaceae* isolation was 78.6% (22 positive swab-cultures) and 7.1% (2 positive swab-cultures) respectively.

During the third week after the injuries 32 swab-cultures were performed, only four (12.5%) showed no growth during this week. Six swab-cultures (21.4%) revealed Gram-positive cocci. The incidence of positive swab-cultures revealed coagulase-negative staphylococci were as low as: 3.5% (1 strain) and *Enterococcus* spp. were 17.9% (5 strains). During this period nonfermentative Gram**-**negative rods were recovered in 25 swab-cultures (89.3% of positive swab-cultures during this period) including the following 75% (21 strains) *A. baumannii* with *Pseudomonas aeruginosa* in the all the rest swab cultures. *Enterobacteriaceae* represented by *Enterobacter* genus, was cultured from the only one wound (3.1% third week swab-cultures).

During the fourth week no growth cultures amounted three (15% of all fourth week swab-cultures). Among Gram-positive microorganisms there were cultured only one *S. aureus* (6.3% of positive swab-cultures) and three strains of *Corynebacterium* (18.8% of positive swab-cultures). During this week nonfermentative Gram-negative rods were still predominant microorganisms cultured of 75% of wounds. The ratio between *Acinetobacter* spp. and *Pseudomonas* spp. appearance was similar to the third week. There were three strains of *Enterobacter* genus (18.8% of positive swab-cultures) cultured during this period.

Five (35.6%) of the 14 swab cultures during fifth week revealed no growth. Gram-positive microorganisms were represented by *Corynebacterium xerosis* (22.2% of positive swab-cultures). *S. aureus* was isolated from the one wound. Nonfermentative Gram negative rods were cultured from 66.7% positive swab-cultures (6 strains).

Antibiotic susceptibility profiles of Gram-negative and Gram positive bacteria isolated from wounds are shown in Additional file 1: Table S1 and Additional file 2: Table S2 respectively.

## Discussion

The battle-field wounds microbiology depends on surgical wound management, interventions such as antimicrobial therapy or infection control strategies, nosocomial transmission in facilities along evacuation route. The trends in changes of the battle-field wounds microbiology, complications after wound contaminations, and associated outcomes in the wound healing are not adequate characterized and defined. Environmental factors, severity and condition of soft tissue injury in each time specified period crucially influence on wound contamination and not always lead to development of wound infection [8]. During the Russo-Turkish War, the first time hospitals were recognized as the places where wound infection developed most often. This fact was later confirmed by microbiological cultures during Vietnam War [9]. Despite immediate wound care, initiation of antimicrobial agents, adequacy of initial wound debridement, definitive surgical care, infectious complications remain the major cause of morbidity. Ability to manage wound infections became more challenging as wound often colonized or infected with multidrug resistant bacteria [10]. Time-specified microbiological surveillance of modern military conflicts war wounds is important for evaluating the effectiveness of current wound management and, in a broader sense, the knowledge of complex relationships between microbiome, human and wound healing.

Analysis of the results of bacteriological researches of war wounds sustained in the conflict in eastern Ukraine in 2014–2015 is consistent with certain previous patterns established during the World War II. However, microbial flora of modern war wound has undergone significant changes. In our study we find that epidemiology of wound infection changes with the time after injury. On the first week after injury the number of no grows swab cultures was the highest (32.1%). The incidence of swab cultures showed no growth decrease during second and third week to 20 and 12.5% respectively. Despite fact, that data reflect clear trend in decreasing incidence of no grows swab cultures the difference is not statistically significant, p ˂ 95%. This pattern established from the time of Vietnam War and confirmed during Soviet-Afghan War and Operation Enduring Freedom. However, the incidence of bacterial isolation from war wounds in studies performed at US military medical facilities significantly lower (30–40%) which can be explained by the difference in initial treatment, in time before injury and first surgical debridement, and methodological approaches used to recover bacteria from wounds [4].

The most common bacterial isolates cultured were Gram-positive bacilli 42.1% (*B. cereus*—36.8%; *C. xerosis*—5.3%) followed by *Staphylococci ssp.* representing 13 strains (37.1%) during first week. The role of *B. cereus* in wound infections in postsurgical patients or wounds subsequent to trauma has also been well defined [11]. Starting with the second week bacterial microflora of the wounds dramatically shifts with increasing recovery of Gram-negative rods. Gram-negative rods were present in 85.7%, on a per swab-culture basis. Nonfermentative Gram negative rods were cultured with highest incidence 78.6% and continue to grow to 89.3% in the third week. Throughout the study *Acinetobacter* species were still predominant microorganisms. The incidence of *Enterobacteriaceae* family microorganisms recovery did not exceed 11.1% during the entire study. The incidence of Gram-positive microorganism’s growth fell after the first week and increased after third week by *Staphylococci* spp. and Gram-positive bacillus genus *Corynebacterium* (18.8%). The incidence of polymicrobial wound cultures increased from two (10.5%) cultures on week number 1 to 6 (21.4%) on second post-injury week. The most often participant of microbial associations of the first week were Gram-positive *Staphylococcus* spp. with *B. cereus*. Microbial mixtures of second and third week consisted of *A. baumannii* with *Enterobacteriaceae* or other nonfermentative Gram negative rods with *Enterococcus* spp. We consider increasing numbers of no growth swab cultures (up to 35.7% on the fifth week) as an evidence of the successful wound healing.

The most common wound pathogens recovered from cultures with polymicrobial growth during Soviet-Afghan War significantly differs from our results. During Soviet-Afghan War, the *Staphylococcus* genus and family *Enterobacteriaceae* dominated in the wounds. There were no *Acinetobacter* species recovered as well as no polymicrobial cultures consisted of only Gram-negative rods [12]. However, our findings are similar to results described in recent conflicts, such as OIF/OEF [13]. Differences in terrain conditions of OIF/OEF (here prevail sands) and conflict in eastern Ukraine (here prevail soil) had low influence on microbiological characteristics of battle-wounds. At least in 2 weeks at the definitive level of medical care gram negative rods and particularly *A. baumannii* predominated. Despite the differences in wound management and antibiotic strategies between Ukrainian and US medical system similarity in microbiological characteristics of battle-wounds looks like a global challenge. That fact that those shifts in wound microbiology developed during patient’s stay in the same medical facility lend further support to the proposition that these changes are nosocomially related [4, 14]. Similar data about microbiological specter of battle-wound which we receive from different parts of the Globe, made us think wider about this problem. Possibly, these microbiological changes in the wounds depend not only on echelon care of military wounded personnel, but are consequence of world antimicrobial practice.

Our study has some limitations. First, there were five similar Ukrainian Military Medical Clinical Centers, where wounded patients received similar surgical care. This study describes microbiological findings in only MMCC CR. The types of microorganisms, which were recovered from the wounds after second week after injury here and which reflect nosocomial transmission in MMCC CR could differ in another hospitals. Our findings deal with observable colonization and are limited by sensitivity of swabbing as a culture method and assessed only for presence of aerobic bacteria. Also with this method we were unable to perform microbiological quantification.

The associations of bacteria recovered from contemporary battle wounds change. It is representing different predominant contaminants at different time intervals after injury. Comparing with the earlier war conflicts, predominant wound microflora is shifting in favor nonfermentative Gram negative rods genus *Acinetobacter* and *Pseudomonas*. Modern intensive care methods, medication, surgical debridement, VAC-therapy significantly influence on microenvironment of wound healing. In our study we observed bacteria recovery from wounds during proliferation phase. These wounds had no pure inflammation signs and were free of devitalized tissues. The fact of wound colonization by typical nosocomial contaminants with successful wound closure put in question strategy of complete eradications of microbes from wound. Actually our study replicate acute wound colonization studies that were carried out in previous war conflicts by Soviet and US scientists. However, our data about wound colonization is new for specific geographic region such as Ukraine and for a new type of conflict as a “Hybrid war”.

## Conclusions

Any wound is at some risk of becoming infected. In the event of infection, a wound fails to heal, the patient suffers increased trauma, treatment costs rise, and general wound management practices become more resource demanding. Determining the microorganisms which colonize battle wounds and cause wound infection is paramount as this information can help to treat such infections or even more changes infection control strategies. The fact of shifting in wound microbiology in the favor of bacteria responsible for healthcare-associated infections such like *S. aureus*, *Enterococcus* spp., *Enterobacteriaceae*, *P. aeruginosa* and *Acinetobacter* spp. support to the proposition that these changes are nosocomially related [4, 14]. For Ukrainian military medicine, this study is the first time-specified assessment of battle wound colonization from the World War II.

## Additional files

**Additional file 1: Table S1.** Antibiotic susceptibility profiles of Gram-negative bacteria isolated from wounds.

**Additional file 2: Table S2.** Antibiotic susceptibility profiles of Gram-positive bacteria isolated from wounds.

## Abbreviations

VAC: vacuum assistance closure
MMCC CR: Military Medical Clinical Center of Central Region
